# Angiotensin-converting enzyme inhibitors of *Bothrops jararaca* snake venom affect the structure of mice seminiferous epithelium

**DOI:** 10.1186/s40409-015-0030-y

**Published:** 2015-08-04

**Authors:** Carlos Alberto-Silva, Joyce M. Gilio, Fernanda C. V. Portaro, Samyr M. Querobino, Antonio C. M. Camargo

**Affiliations:** Center for Natural and Humanities Sciences (CCNH), Federal University of ABC (UFABC), R. Santa Adélia, 166, Santo André, SP CEP 09210-170 Brazil; Center for Applied Toxinology, Butantan Institute, São Paulo, SP Brazil; Laboratory of Immunochemistry, Butantan Institute, São Paulo, SP Brazil

**Keywords:** *B. jararaca*, Bradykinin-potentiating peptides, Angiotensin-converting enzyme, Seminiferous epithelium, Spermatogenesis

## Abstract

**Background:**

Considering the similarity between the testis-specific isoform of angiotensin-converting enzyme and the C-terminal catalytic domain of somatic ACE as well as the structural and functional variability of its natural inhibitors, known as bradykinin-potentiating peptides (BPPs), the effects of different synthetic peptides, BPP-10c (<ENWPHQIPP), BPP-11e (<EARPPHPPIPP), BPP-AP (<EARPPHPPIPPAP) and captopril were evaluated in the seminiferous epithelium of male mice.

**Methods:**

The adult animals received either one of the synthetic peptides or captopril (120 nmol/dose per testis) via injection into the testicular parenchyma. After seven days, the mice were sacrificed, and the testes were collected for histopathological evaluation.

**Results:**

BPP-10c and BPP-AP showed an intense disruption of the epithelium, presence of atypical multinucleated cells in the lumen and high degree of seminiferous tubule degeneration, especially in BPP-AP-treated animals. In addition, both synthetic peptides led to a significant reduction in the number of spermatocytes and round spermatids in stages I, V and VII/VIII of the seminiferous cycle, thickness of the seminiferous epithelium and diameter of the seminiferous tubule lumen. Interestingly, no morphological or morphometric alterations were observed in animals treated with captopril or BPP-11e.

**Conclusions:**

The major finding of the present study was that the demonstrated effects of BPP-10c and BPP-AP on the seminiferous epithelium are dependent on their primary structure and cannot be extrapolated to other BPPs.

## Background

Two isoforms of the angiotensin-converting enzyme (ACE) have been reported in different animals, and denominated testicular isoform (tACE) and the somatic (sACE) isoform [1]. The structural analysis of both isoforms indicates that tACE harbors only the C-site of sACE, which is directly associated with male fertility [1–7]. Furthermore, tACE is expressed in germ cells in spermatids and spermatozoa, but not in Sertoli, Leydig or other somatic cells, thereby suggesting that tACE is involved in spermiogenesis [5, 6]. In contrast, sACE (EC 3.4.15.1) is a peptidase of the cell membrane of endothelial cells that has two catalytic sites, the amino-terminal (N-site) and carboxyl-terminal (C-site) [8, 9]. The N-site metabolizes a peptide hormone (Ac-Ser-Asp-Lys-Pro) associated with the negative regulation of hematopoiesis [10]. The C-site converts angiotensin I (Ang I) into angiotensin II (Ang II), which is a hypertensive peptide and promotes the degradation of bradykinin, a hypotensive peptide [11, 12].

The first described natural inhibitors of sACE were bradykinin-potentiating peptides (BPPs) derived from snake venom. BPPs are oligopeptides rich in proline, which display bradykinin-potentiating activity, range from 5 to 14 amino acid residues and display a common pyroglutamic acid (<E) residue at the N-terminal position and a proline residue at the C-terminal position [13]. We have demonstrated that synthetic BPPs display remarkable functional differences despite their high amino acid sequence similarities [13]. Some BPPs specifically inhibit the C-site, such as BPP-10c, while others seem to be either selective for the N-site or poor ACE inhibitors [14]. Interestingly, biotransformation studies of BPPs with different structural activities and the characterization of metabolites in mouse urine have indicated that the diverse biological functions of each BPP could be mediated via the production of different metabolites due to different interactions with alternative targets [15].

The effect of BPPs derived from *Bothrops jararaca* snake venom on spermatogenesis in mice has been characterized by our group. Interestingly, we have demonstrated that BPP-10c, a potent selective C-domain inhibitor of sACE and not captopril, modified spermatogenesis in male Swiss mice treated for 15 consecutive days with a single dose of BPP-10c (4.7 μmol/kg/d) by intraperitoneal administration [16].

Intratesticular (i.t.) injection has been employed to characterize the initial effect ofanti-spermatogenic molecules, as it optimizes the injected dose and facilitates the entry of the molecule of interest in the testis [17]. Thus, considering the structural and functional particularities of BPPs we selected the different peptides [BPP-10c (<ENWPHQIPP), BPP-11e (<EARPPHPPIPP), BPP-AP (<EARPPHPPIPPAP), (inv)BPP-10c (PPIQPHPWNE, containing the inverted BPP-10c sequence)] and captopril for the assessment of their effects on the dynamics and structure of the seminiferous epithelium in mice following i.t. injection.

## Materials and methods

### Reagents and synthesis of BPPs

All chemicals were of analytical reagent grade (purity higher than 95 %) and purchased from Calbiochem-Novabiochem Corporation (USA), Merck (Germany) and Sigma-Aldrich Corporation (USA) for peptide synthesis. Captopril was purchased from Sigma Chemical Company (USA). BPP-10c, BPP-11e, BPP-AP and (inv)BPP-10c tested in present study were synthesized via solid phase peptide synthesis applying the Fmoc (9-fluorenylmethyloxycarbonyl) strategy [13–16]. The synthetic peptides were purified by preparative reversed-phase chromatography (reversed-phase HPLC), whereas the purity and identity of the peptide were confirmed by MALDI-TOF mass spectrometry on an Ettan MALDI-TOF/Pro instrument (Amersham Biosciences, USA). A purity higher than 95 % was achieved for all peptides.

### Animals

Mature male Swiss mice housed with sanitary barriers from the Central Animal Facility of the Butantan Institute (São Paulo, Brazil) were authorized for use by the Ethics Committee of the Butantan Institute (protocol n° 369/07). The specimens (body weight 30 to 35 g; age 7 to 8 weeks) received standardized mouse chow (Nuvital Nutrientes Ltda, Brazil) *ad libitum* and were housed four animals per cage, with a 12-h light/dark photoperiod and constant exhaust ventilation (Alesco®, Brazil) in the conventional mammal experimentation animal facility of the Center for Applied Toxinology (CAT/Cepid), Butantan Institute.

### Intratesticular injection of BPPs and captopril

Twenty-five mice were divided into six groups (G1-G6) and anesthetized with Ketamine® and Xylazine® (3:1) at a dose of 174 μg and 11.5 μg per gram of body mass, respectively. The animals were submitted to an abdominal incision (median retro-umbilical longitudinal laparotomy), and the right and left testes were exposed in the abdominal cavity. The agents were injected directly into the testicular parenchyma of the left testis of each animal (two sites per testis); approximately 10 μL of synthetic peptide or drug [BPP-10c, BPP-11e, BPP-AP, (inv)BPP-10c or captopril diluted in 0.91 % w/v aqueous sodium chloride solution at a concentration of 120 nmol/dose] or vehicle only (control group). Each sample was administered using a 0.5-mL syringe and a 30-gauge needle (Ultra-Fine Short Needle, BD, Canada) as detailed by Chung et al. [17].

Following the surgery, the animals were maintained in the animal facility for seven days and then euthanized by CO_2_ asphyxiation. The left testes were collected for morphological and morphometric analysis, although the specimens were examined with the researchers blinded to knowledge of the treatment group. Additionally, morphological analysis of the right testes without treatment was also carried out to assess possible changes caused by the treatment procedures performed on the left testis in each animal. All treatments and experiments were performed in duplicate.

### Processing of the tissue

The testes were immersed in Bouin fixing solution (4 % formaldehyde with picric acid) (v/v) for eight hours, dehydrated in increasing concentrations of alcohol (70 % to 95 %) (v/v) and embedded in Paraplast® (Sigma Chemical Company, USA). The histological slices (4 μm in thickness) were performed on an automatic microtome (Leica RM 2155; Germany) with a steel blade and placed in a water bath (42 °C) for placement on glass slides. Following deparaffinization with xylol and absolute alcohol-xylol (1:1, v/v), the slices were hydrated in decreasing alcohol solution and stained with either hematoxylin and eosin or Mallory’s trichrome stain for morphological analysis of the seminiferous epithelium.

The periodic acid-Shiff (PAS) with Harris hematoxylin histochemical method was used to determine the stages of the seminiferous epithelium cycle. After staining, the slices were dehydrated and mounted, and the slipcover was attached to the slide with Dammar gum. The preparations were examined using a photomicroscope (Axioskop 2, Zeiss, Germany), and the images were captured with a Pixera digital camera system (Pixera Corporation, USA) attached to the photomicroscope and a microcomputer (Intel® Pentium®) using the software Adobe Photoshop version 7.0.1 (Adobe Systems, USA).

### Determination of morphological parameters of the seminiferous epithelium

The degree of tubular degeneration for each treatment group was analyzed double-blindly, to evaluate the influence of intratesticular administration of each agent in the morphology of the seminiferous tubules. The tubules were classified into four categories: normal tubules (T1); hypospermatogenic tubules characterized by a decrease in the thickness of the seminiferous epithelium, which contained all germ cell types and occasionally sloughed germ cells (T2); arrested maturation, in which the seminiferous epithelium showed hypospermatogenic areas joined to others with arrested maturation of germ cells, predominantly at the level of spermatocytes (T3); and tubules containing spermatogonia and Sertoli cells or Sertoli-cell-only tubules (T4). For this purpose, three 4-μm-thick sections of each testis, which were randomly selected, were used and 100 tubular cross-sections were examined from each section. Additionally, the morphological aspects of the intertubular compartment, in particular Leydig cells, blood vessels, lymph vessels, fibroblasts, macrophages, and mast cells, were also analyzed.

### Determination of morphometric parameters of the seminiferous epithelium

Stages I, V, VII/VIII and XII of the seminiferous epithelium cycle, representing the beginning, middle and end of this cycle, respectively, were selected for the blind analysis of cell numbers and morphometric parameters of the seminiferous epithelium [18]. Each stage was identified based on acrosome development and morphology of the nucleus of the spermatids during differentiation [19]. For morphometric analysis, eight circular or nearly circular seminiferous tubules were randomly selected in each stage studied per testis. The images captured were analyzed using the software ImageJ (National Institutes of Health, USA) to assess the diameter of the seminiferous tubules (mm), thickness of the seminiferous epithelium (mm) and diameter of the seminiferous tubule lumen (mm).

### Quantitative analysis of the cells in the seminiferous epithelium

The images used for morphometric analysis were also utilized for quantitative analysis. The images were analyzed with the aid of Adobe Photoshop version 7.0.1 (Adobe Systems, USA), with a reticulated grid of 1575 points at 66.7 % magnification. Only the nuclei of the cells in the intersection points were counted. The following cell types were counted for each stage (I, V, VII/VIII, and XII) of the seminiferous epithelium cycle: type A spermatogonium, type B spermatogonium, preleptotene spermatocyte, zygotene spermatocyte, meiotic figures, secondary spermatocytes, pachytene spermatocyte, round spermatid and Sertoli cells.

### Total support capacity of each Sertoli cell

Assessment of the total support capacity was performed with the data derived from the quantitative analysis, dividing the total number of germ cells by the total number of Sertoli cells per stage, based on the protocol described by Russell and Peterson [20].

### Statistical analysis

The software GraphPad Prism (version 4.0; GraphPad Software, Incorporation) was used for statistical analyses. Statistical significance for all experiments was analyzed using one-way analysis of variance (ANOVA) followed by the Tukey test as a *post hoc* test. Values represent the mean ± SEM, and *p* < 0.05 was considered statistically significant.

## Results

### Morphological analysis of the seminiferous epithelium

**Fig. 1:**
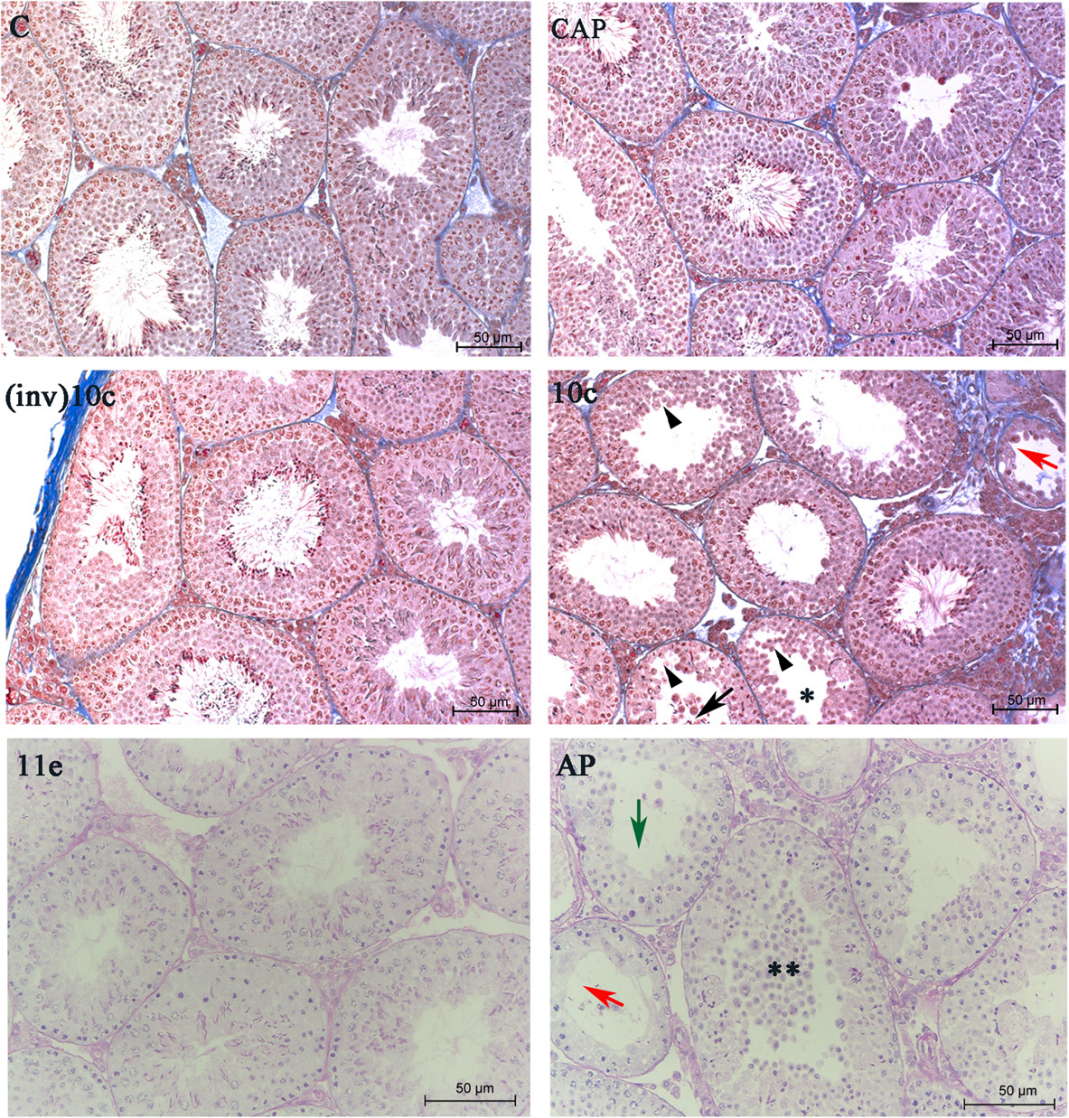
Effects of angiotensin-converting enzyme (ACE) inhibitors on morphology of the seminiferous epithelium of the mouse testis. Seminiferous tubules of the right testis of control animals treated with saline solution (**C**) and the left testis of mice treated with captopril (**CAP**), (inv)BPP-10c [**(inv)10c**], BPP-10c (**10c**), BPP-11e (**11e**) or BPP-AP (**AP**). Normal structure of tubules [**C**, **CAP**, **(inv)10c**, and **11e**], germ cells in the lumen oftubules (**10c**, black arrow), degenerative tubule (**10c** and **AP**, red arrow), ruptures in the epithelium (**10c**, arrowheads), absence of round spermatids in stage V of the seminiferous epithelium cycle (**10c**, asterisk), round spermatids in the adluminal compartment (**AP**, double asterisk) and tubules with few germ cells, green arrow). Staining: Mallory’s Trichrome [**C**, **CAP**, **(inv)10c** and **10c**] and PAS-Hematoxylin (**11e** and **AP**)

The analysis of the testes of mice treated with saline solution, captopril, and (inv)BPP-10c indicated that these molecules do not alter the morphological characteristics of the seminiferous epithelium with regard to the arrangement of germ cells and integrity of the tubule and interstitial compartment, considering the seminiferous epithelium cycle (I-XII) [Fig. 1, panels C, CAP, (inv)10c]. In contrast, the seminiferous epithelium of animals treated with BPP-10c displayed an absence of elongated spermatids in the seminiferous tubules at stages I, V and VII/VIII of the seminiferous epithelium cycle as well as the presence of developing germ cells, immature germ cells in the seminiferous tubule lumen, discontinuity of the seminiferous epithelium caused by the displacement of germ cells of the adjacent epithelium and ruptures in the seminiferous epithelium (Fig. 1, panel 11e).

Interestingly, BPP-11e did not cause alterations in the seminiferous epithelium compared with the control group (Fig. 1, panel 11e). Moreover, BPP-AP demonstrated more intensive impairment of spermatogenesis in the seminiferous tubules (Fig. 1, panel ap) compared with that observed following BPP-10c treatment. Hypospermatogenic seminiferous tubules with Sertoli cells only were identified, along with an intensive loss of germ cells in the lumen, an absence of elongated spermatids at stages I, V, VII/VIII and immature germ cells in the lumen of the seminiferous tubules (Fig. 1, panel ap). Additionally, no changes were observed in the intertubular compartment of the animals treated with BPPs tested or captopril.

### Degree of tubule degeneration

The degree of tubule degeneration (Table 1), according to the four-category scale and based on the mean of tubule types present in each group, was significantly different in the BPP-10c and BPP-AP groups compared to the control group. Meanwhile, no significant difference was found between the control groups (right testis and left testis) (data not shown) or between the BPP-11e, (inv)BPP-10c or captopril groups and the control group. Additionally, arrested maturation of the tubules was observed only in the BPP-10c and BPP-AP treatment groups, while tubules containing only spermatogonia and Sertoli cells were detected in the BPP-AP treatment group.

**Table 1:** Semi-quantitative histological assessment of seminiferous tubules of mouse testes

Treatment	T1 (%)	T2 (%)	T3 (%)	T4 (%)
C	92.33 ± 1.20	10.33 ± 0.89	0	0
CAP	94.32 ± 1.02	5.66 ± 1.12	0	0
10c	49.67 ± 0.88^*^	38.32 ± 1.43^*^	12.01 ± 0.57^*^	0
(inv)10c	92.65 ± 0.86	7.36 ± 0.78	0	0
11e	91.67 ± 1.48	8.39 ± 0.90	0	0
AP	37.69 ± 2.35^*^	55.63 ± 3.06^*^	5.00 ± 0.53^*^	1.62 ± 0.23^*^

The data are presented as the mean ± SEM. Types of degeneration tubular in each treatment [CAP, 10c, inv(10c), 11e and AP] were compared to the control (C), and significant differences as assessed through one-way ANOVA followed by the Tukey test as a *post hoc* test (* *p* < 0.05). Normal tubules (T1); hypospermatogenic tubules (T2); arrested maturation tubules (T3); tubules containing spermatogonia and Sertoli cells or Sertoli-cell-only tubules (T4). C, control; CAP, captopril; (inv)10c, containing the inverted BPP-10c sequence; 10c, BPP-10c; 11e, BPP-11e; AP, BPP-AP

### Morphometric analysis of the seminiferous epithelium

**Fig. 2:**
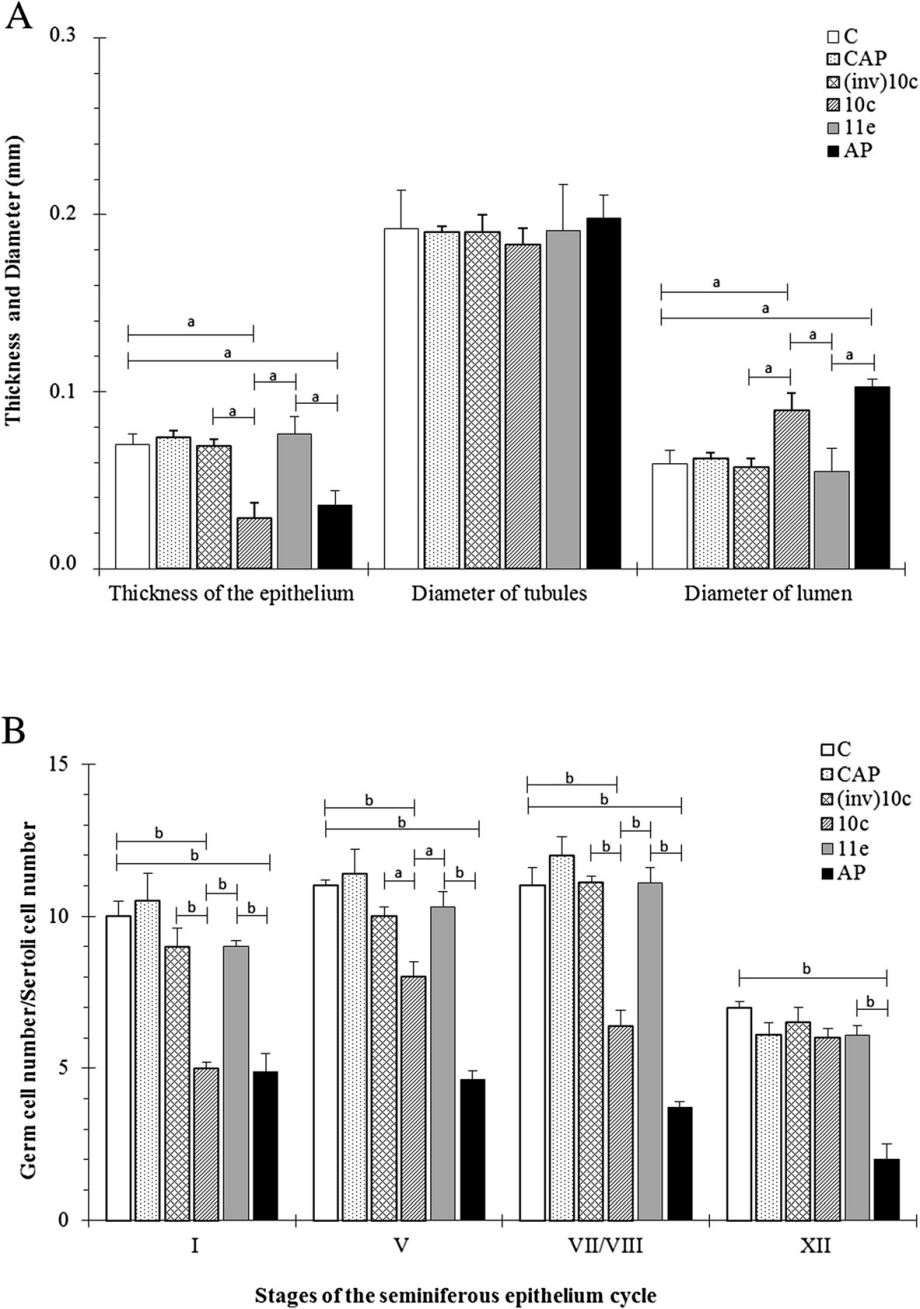
Morphometric aspects of the seminiferous tubules in mice treated with angiotensin-converting enzyme (ACE) inhibitors. **a** Thickness of the epithelium and diameter of tubules and lumen of the seminiferous tubules of control and treated animals. **b** Total support capacity of Sertoli cells during stages I, V, VII/VIII and XII of the seminiferous epithelium cycle of control and treated animals. The data are presented as the mean ± SEM. The various letters indicate significant differences between the analyzed groups as determined via one-way ANOVA followed by the Tukey test as a *post hoc* test (a, *p* < 0.001; b, *p* < 0.0001). C, control; CAP, captopril; (inv)10c, containing the inverted BPP-10c sequence; 10c, BPP-10c; 11e, BPP-11e; AP, BPP-AP

BPP-10c treatment displayed a reduction in the thickness of the seminiferous epithelium and an increase in the diameter of the seminiferous tubule lumen compared with the (inv)BPP-10c, BPP-11e, captopril and control groups (Fig. 2 –a). Treatment with BPP-AP led to a significant reduction in the thickness of the seminiferous epithelium and an increase in the diameter of the seminiferous tubule lumen compared with the BPP-11e, captopril and control groups (Fig. 2 – a). No alteration was detected in the diameter of tubules in all treatment groups.

### Total support capacity of each Sertoli cell

Treatment with BPP-10c led to a reduction in total support capacity of each Sertoli cell in stages I, V and VII/VIII compared with the (inv)BPP-10c, BPP-11e, captopril and control groups (Fig. 2b). Likewise, treatment with BPP-AP also led to a significant reduction in total support capacity of each Sertoli cell in stages I, V, VII/VIII and compared with the control, captopril and BPP-11e groups (Fig. 2b), and specifically in stage XII.

### Quantitative analysis of the cells in the seminiferous epithelium

**Fig. 3:**
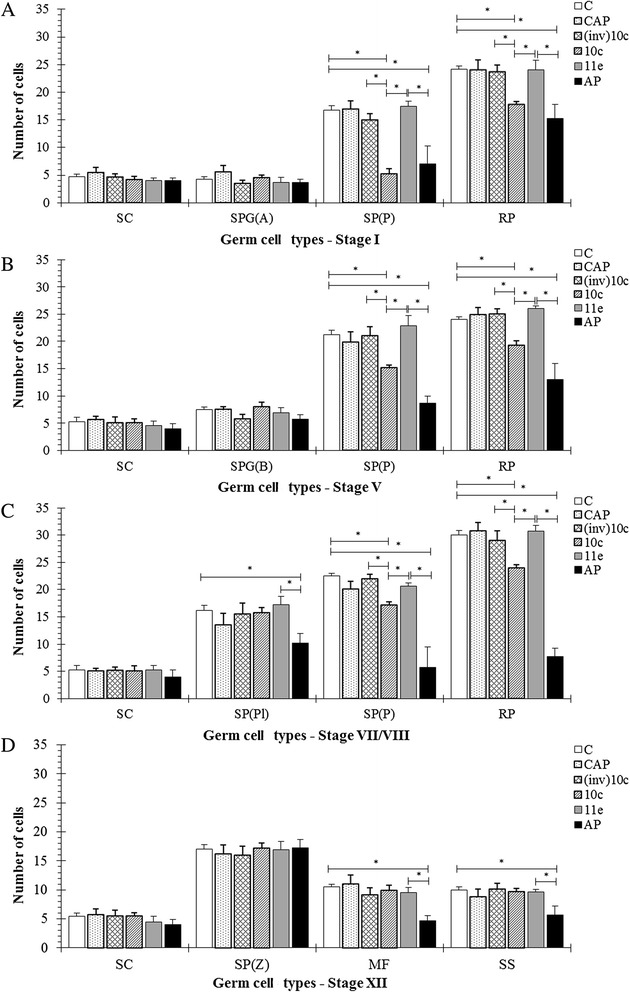
The effects of angiotensin-coverting enzyme (ACE) inhibitors on the number of germ cell types in the seminiferous epithelium cycle. The germ cells present at stages (**a**) I, (**b**) V, (**c**) VII/VIII, and (**d**) XII of the seminiferous epithelium cycle were counted in all conditional treatments. The data are presented as the mean ± SEM; various superscripts designate significant differences between the analyzed groups as assessed using one-way ANOVA followed by the Tukey test as a *post hoc* test (a, *p* < 0.01; b, *p* < 0.001; c, *p* < 0.0001). SC, Sertoli cell; SPG(A), type A spermatogonium; SPG(B), type B spermatogonium; SP(Pl), preleptotene spermatocyte; SP(Z), zygotene spermatocyte; SP(P), pachytene spermatocyte; MF, meiotic figures; SS, secondary spermatocyte; RP, round spermatid; C, control; CAP, captopril; (inv)10c, containing the inverted BPP-10c sequence; 10c, BPP-10c; 11e, BPP-11e; AP, BPP-AP

Spermatocytes in pachytene and round spermatid numbers were significantly reduced in stages I, V and VII/VIII of the seminiferous epithelium cycle in the BPP-10c and BPP-AP groups (Fig. 3 – a, b, c) compared with the control, captopril, (inv)BPP-10c and BPP-11e groups. BPP-AP also led to a reduction in the number of preleptotene spermatocytes and round spermatids in stages VII/VIII compared with the BPP-11e and control groups (Fig. 3 – c). Moreover, only BPP-AP led to a reduction in the number of meiotic figures and secondary spermatocytes at stage XII, which is the end of the seminiferous epithelium cycle (Fig. 3 – d). No differences were detected in the number of Sertoli cells in all treatment groups and all stages tested.

## Discussion

The major finding of the present study was the observation that the effects of BPP-10c and BPP-AP on the seminiferous epithelium are dependent on their primary structure and cannot be extrapolated to other BPPs. Interestingly, captopril and BPP-11e, which are also sACE inhibitors, did not show any changes in the structure of the seminiferous epithelium. Considering the structural similarity between the C-terminal sites of sACE and tACE [] and the observation that male tACE “knockout” mice are hypofertile, ACE inhibitors may reduce male fertility [21–23]. Captopril, lisinopril and enalapril have been reported to act as tACE inhibitors *in vitro* [21, 24, 25]. Likewise, BPP-5a (<EKWAP) and BPP-9a (<EWPRPQIPP), which are natural sACE inhibitors, are also tACE inhibitors *in vitro*, acting at the nanomolar level [26, 27]. Nevertheless, it has been demonstrated that captopril and its derivatives did not affect tACE activity *in vivo*, thereby suggesting that these drugs do not cross the BTB, which would explain the absence of reports concerning the adverse effects of ACE inhibitors on testicular function [28, 29]. However, we have demonstrated in previous studies that BPP-10c, the most potent and selective sACE C-domain inhibitor, modified spermatogenesis in mice treated by intraperitoneal injection without affecting BTB permeability or the distribution of claudin-1, a protein found at the site of the BTB [16]. It was very interesting to find that the effects are dependent on the primary molecular structure of BPP-10c, since no morphological or morphometric alterations in the seminiferous epithelium were found in the mice treated with (inv)BPP-10c, that contains the inverted BPP-10c sequence.

Experimental evidence from our group has demonstrated that BPPs interact *in vivo* with other molecular targets in addition to sACE [13]. We have demonstrated that BPP-10c is capable of positively modulating the catalytic activity of argininosuccinate synthase (AsS) *in vitro* and *in vivo* and consequently induces NO production in endothelial cells [30]. AsS is a ubiquitous enzyme that can be detected in the lungs, brain and testes [31]. AsS catalyzes the conjugation reaction of citrulline with aspartate, thereby generating argininosuccinate with the uptake of adenosine triphosphate (ATP). Argininosuccinate is a substrate of argininosuccinate lyase (AsL), which converts it into L-arginine, a substrate of nitric oxide synthase (NOS) in the production of nitric oxide (NO) [31]. NO is one of the mediators that control the opening and closing of the junctional complexes in the seminiferous epithelium via the NO/soluble guanylate cyclase/cGMP protein kinase G/b-catenin signaling pathway [32]. This mechanism contributes to the control of migration of developing germ cells from the basal compartment to the adluminal compartment seminiferous epithelium [33].

BPP-10c caused ruptures in the seminiferous epithelium and loss of germ cells in the lumen of the seminiferous tubules, typical alterations in the dynamics of junctional complexes (occludin and adherens junctions) in the seminiferous epithelium [32–34]. Thus, as BPP-10c caused ruptures in the epithelium and acts as a positive modulator of AsS, these peptides can increase the levels of NO in the testis, thereby causing the opening of the junctional complexes and the appearance of immature germ cells in the seminiferous tubule lumen. However, new experiments should be designed to confirm this hypothesis.

We also show that the presence of immature germ cells in the lumen of the seminiferous tubules and discontinuity and ruptures of the seminiferous epithelium were more evident in the BPP-AP treatment group than in the BPP-10c group. Yet another interesting result was the effects of BPP-11e (<EARPPHPPIPP) and BPP-AP (<EARPPHPPIPPAP) on spermatogenesis in mice. We demonstrated that the presence of the alanine and proline amino acids in the C-terminal portion of BPP-AP was essential for the effects observed, which once again demonstrates that the varying effects of different BPPs on spermatogenesis in mice is attributed to their primary structure.

BPP-AP and BPP-11e were identified in venom derived from the snake *Bothrops jararacussu* using the inactive zinc metalloprotease thimet oligopeptidase (EP24.15) as a peptide bait to isolate specific bioactive peptides from complex mixtures [35]. BPP-AP displayed an inhibitory effect on sACE (0.035 μM) and EP24.15 (1.6 μM), which are lower than the BPP-11e values observed (0.084 μM and 9.5 μM, respectively) [35]. EP24.15 is found predominantly in the neuroendocrine–gonadal axis, where it is implicated in the progression of spermatogenesis [36]. However, the discrete differences in *Ki* between the two BPPs *in vitro* are insufficient to explain the intensive effects of BPP-AP on spermatogenesis in mice and the absence of alterations following BPP-11e treatment *in vivo*. In contrast, *in vivo* assays have suggested that BPP-AP, but not BPP-11e, can induce the release of vasodilatation mediators and increase the expression of integrins in both leukocytes and on the surface of endothelial cells, thereby leading to adhesion and extravasation of rolling leukocytes [35]. Similarly, we also demonstrate that only BPP-AP promotes changes in morphology of the seminiferous epithelium, which may be associated with the minor structural differences between these peptides.

We have previously shown that BPP-10c is internalized by HUVEC, HEK293 and C6 cells in different experimental conditions [16, 30, 37]. These results are not surprising considering that BPPs are proline-rich peptides, a feature that endows these molecules with the properties of cell-penetrating peptides and resistance to proteolysis [27]. These data indicate that these peptides may be internalized by Sertoli cells; however, the different effects observed in the seminiferous epithelium of mice treated with different proline-rich peptides and the hypothesis that these peptides are internalized by Sertoli cells are not sufficient to explain the pathogenic variations, as BPP-11e did not demonstrate effects in the seminiferous epithelium. BPPs have shown remarkable functional differences, despite their high amino acid sequence similarities; furthermore, new targets that shed light on their biological activities have been identified [13].

We have tested a number of BPPs with different structure activities [i.e., BPP-5a (<EKWAP), BPP-7a (<EDGPIPP), BPP-9a (<EWPRPQIPP), BPP-10c (<ENWPHQIPP) and BPP-12b (<EWGRPPGPPIPP)] and studied their stability when exposed to endogenous animal proteolytic enzymes in mice [16, 38]. Sequence analysis of urinary metabolites of BPPs, performed via MALDI-TOF mass spectrometry, generated the following findings: BPP-7a showed increased resistance to proteolytic cleavage; BPP-5a was totality metabolized into < EKW; BPP-9a was identified in the intact form as well as the < EWPRP and < EWPRPQIP forms; BPP-10c was found in its intact form and as a unique metabolite (<ENWPHQIP); and BPP-12a proved to be highly susceptible to hydrolysis by proteolytic enzymes [16, 38]. Thus, the results obtained support the hypothesis that the diverse biological functions of each BPP may be mediated by different interactions with alternative targets and not only by sACE inhibition.

In fact, we showed that BPP-10c was capable of positively modulating the catalytic activity of AsS *in vitro* and *in vivo* and consequently induced NO production in endothelial cells, which explains the hypotensive effect independent of sACE inhibition [30]. In the present study, we hypothesized that the presence of the C-terminal portion of BPP-AP was important for producing the effects observed in the seminiferous epithelium compared with BPP-11e, which may be explained by the specificity of interaction with unknown targets.

## Conclusion

The results of the current study suggest that the Chinese version of OBQ-44 is a reliable and valid instrument to assess dysfunction beliefs in Chinese population. It is also useful for researchers to evaluate interventions aiming to reduce dysfunction beliefs. Furthermore, its validity is contributed to the cross-cultural comparison in the future.

### Ethics committee approval

All experimental protocols were performed in accordance with the guidelines of the human use of laboratory animals of Butantan Institute and approved by local authorities (protocol number 369/07).
