# Erratum to: Angiotensin-converting enzyme inhibitors of Bothrops jararaca snake venom affect the structure of mice seminiferous epithelium

**DOI:** 10.1186/s40409-015-0032-9

**Published:** 2015-08-26

**Authors:** Carlos Alberto-Silva, Joyce M. Gilio, Fernanda C. V. Portaro, Samyr M. Querobino, Antonio C. M. Camargo

**Affiliations:** Center for Natural and Humanities Sciences (CCNH), Federal University of ABC (UFABC), R. Santa Adélia, 166, Santo André, SP CEP 09210-170 Brazil; Center for Applied Toxinology, Butantan Institute, São Paulo, SP Brazil; Laboratory of Immunochemistry, Butantan Institute, São Paulo, SP Brazil

Unfortunately, the original version of this article [1] contained an error. The conclusion was included incorrectly. The conclusion has been corrected in the original article and is also included correctly below.

## Conclusion

Overall, the results obtained from the proline-rich snake-venom oligopeptide suggest that the alterations in the structure of the seminiferous epithelium in mice following BPP-10c and BPP-AP treatment, but not treatment with BPP-11e, are dependent on their primary molecular structure. This study offers new perspectives for the elucidation of possible mechanisms involved in the impairment of spermatogenesis by BPP-10c and BPP-AP, thereby providing new insight into the biological features of the snake venom.

## References

[1] Alberto-Silva C, Gilio P, Querobino C. Angiotensin-converting enzyme inhibitors of Bothrops jararaca snake venom affect the structure of mice seminiferous epithelium. J Venomous Anim Toxins Incl Tropical Dis. 2015;21:27. 10.1186/s40409-015-0030-y PMC4524108 26244047

